# OPTIMISM AND PAIN INTERFERENCE IN AGING WOMEN

**DOI:** 10.1093/geroni/igz038.3000

**Published:** 2019-11-08

**Authors:** Stephanie T Judge, Jodi L Clasey, Leslie J Crofford, Suzanne C Segerstrom

**Affiliations:** 1 Department of Psychology, University of Kentucky, Lexington, Kentucky, United States; 2 Department of Kinesiology and Health Promotion, University of Kentucky, Lexington, Kentucky, United States; 3 Departments of Medicine and Pathology, Microbiology, and Immunology, Vanderbilt University School of Medicine, Nashville, Tennessee, United States

## Abstract

Pain limits individuals’ ability to engage in activities that promote well-being. This longitudinal-burst daily diary study tested reciprocal relationships among pain, optimism, pain interference, and activity in older women. Multilevel models tested between- and within-person relationships among these variables. Pain best predicted interference (person: γ001 = .227, SE = .022, p < .0001; wave: γ010 = .267, SE = .014, p < .0001; day: γ100 = .246, SE = .010, p < .0001); optimism best predicted activity (γ002 = .684, SE = .101, p < .0001). In linear regression models, baseline optimism (sr2 = 0.560, p < .0001), less interference (sr2 = 0.064, p < .0001), and more activity (sr2 = 0.015, p = .013) predicted higher end-of-study optimism. Ultimately, more optimistic women were significantly more active than less optimistic women, and less interference and more activity promoted increased optimism, creating a virtuous cycle that enhances well-being among older women.

